# Do the shuffle: Changes in *Symbiodinium* consortia throughout juvenile coral development

**DOI:** 10.1371/journal.pone.0171768

**Published:** 2017-02-09

**Authors:** Hannah G. Reich, Deborah L. Robertson, Gretchen Goodbody-Gringley

**Affiliations:** 1 Department of Biology, Clark University, Worcester, Massachusetts, United States of America; 2 Bermuda Institute of Ocean Sciences, 17 Biological Lane, St. George, Bermuda; Helmholtz-Zentrum fur Ozeanforschung Kiel, GERMANY

## Abstract

Previous studies of symbiotic associations between scleractinians corals and *Symbiodinium* have demonstrated that the consortium of symbionts can change in response to environmental conditions. However, less is known about symbiont shuffling during early coral development, particularly in brooding species. This study examined whether *Symbiodinium* consortia (1) varied in *Porites astreoides* on shallow (10m) and upper mesophotic (30m) reefs, (2) changed during coral development, and (3) influenced growth of juveniles in different environments. *Symbiodinium* ITS2 sequences were amplified using universal primers and analyzed using phylotype-specific primers designed for phylotypes A, B, and C. Adults from both depths were found to host only phylotype A, phylotypes A and B, or phylotypes A, B, and C and the frequency of the phylotype composition did not vary with depth. However, phylotype A was the dominant symbiont that was vertically transmitted to the planulae. The presence of phylotypes B and C was detected in the majority of juveniles when transplanted onto the shallow and upper mesophotic reefs whereas only phylotype A was detected in the majority of juveniles reared in outdoor aquaria. In addition, growth of juvenile *P*. *astreoides* harboring different combinations of *Symbiodinium* phylotypes did not vary when transplanted to different reef zones. However, juveniles reared in *in situ* reef environments grew faster than those reared in *ex situ* outdoor aquaria. These results show that *Symbiodinium* consortia change during development of *P*. *astreoides* and are influenced by environmental conditions.

## Introduction

In recent decades, shallow coral reef communities have experienced large-scale declines driven by anthropogenic and environmental stressors [1–3]. Coral recovery is influenced by the frequency and magnitude of ongoing disturbances as well as the dynamics of population maintenance, recruitment, structure, and size [4]. Population maintenance and recruitment can occur through horizontal connectivity and vertical mixing, allowing for the increase in coral cover during both processes. These processes are shaped by patterns of dispersal and genetic connectivity, which vary with coral reproductive life histories. Broadcast spawning corals that undergo external fertilization, have been found to have high levels of genetic connectivity throughout the Caribbean and Western Atlantic, which may be attributed to high rates of cross-fertilization and a long pelagic dispersal period [5–7]. In contrast, brooding corals that undergo internal fertilization have lower levels of genetic connectivity throughout the Caribbean and Western Atlantic basin [8, 9], making local recruitment and juvenile acclimation crucial for population maintenance.

The brooding coral, *P*. *astreoides* is projected to be an important player on Caribbean and Western Atlantic reefs due to its increase in abundance, despite large-scale declines in coral cover and condition for other scleractinians species [10]. For example, in Curacao, relative abundance of juvenile *P*. *astreoides* (<4 cm in diameter) doubled between 1975–2005 [11]. In addition to an increase in relative abundance, *P*. *astreoides* has resilient qualities such as a genetic basis for thermal resilience [12, 13] and “weedy” life-history traits including its high levels of fecundity and settlement densities [10, 14, 15].

Though its increase in abundance and qualities of resilience positions *P*. *astreoides* to succeed on Caribbean reefs, little is known about *P*. *astreoides* early growth, symbioses during juvenile development, and mechanisms of acclimation to new environments. Knowledge of early growth and juvenile acclimation is essential in assessing population dynamics and recruitment success. To date, few studies have examined early growth and acclimation of juvenile brooding corals. When done so, these processes were monitored by collecting juveniles from the reef with subsequent experimentation [16–19] or by lab-rearing juveniles [20]. Early growth and symbioses has been more broadly examined in juveniles of broadcasting corals by inoculating aposymbiotic larvae with various strains of symbionts from the genus *Symbiodinium* and monitoring growth in lab or on the reef [21–26]. Having a deeper understanding of symbioses during juvenile growth and acclimation to differing environmental conditions is crucial to assessing *P*. *astreoides* population dynamics as it continues to succeed in the Caribbean-Western Atlantic region. Likewise, determining how these patterns differ in laboratory reared juveniles (*ex situ*) compared to those reared in the natural reef environment (*in situ*) is critical for interpretation of laboratory based studies on juvenile development and symbiotic relationships.

Important to these processes are photosynthetic symbionts from the genus *Symbiodinium*. These photosynthetic symbionts can provide the host coral with up to 100% of their energy requirements, making them crucial for coral growth and survival [27]. Currently there are nine genetically identified clades (A-I) of *Symbiodinium* that exhibit unique physiological traits and functional diversity [27, 28]. Harboring different combinations of *Symbiodinium spp*. and shuffling symbiont consortia can lead to enhanced coral fitness, growth, survival, and thermotolerance [22, 29–34]. Symbiont switching, or horizontal acquisition of *Symbiodinium*, leading to the accumulation of more thermotolerant symbionts (clade D *Symbiodinium*) has been found to be a strategy for environmental acclimation in adult corals during moderate warming events [14, 30, 34–36] and during recovery from bleaching events [33]. Alternatively, corals harboring clade C *Symbiodinium* often exhibit faster growth [31, 37] because clade C *Symbiodinium* translocates more carbon to the host relative to other *Symbiodinium* clades [21, 38]. However, these tradeoffs are compromised in the presence of thermal stress, where differences in growth of corals harboring different *Symbiodinium* clades is negligible [39]. Though it releases less carbon to the host in comparison to *Symbiodinium* clade C, members of clade A are thought to be highly productive [38], have a pigment profile which suggests potential for bleaching resistance [40], and possess pathways associated with increased photoprotection and survival in shallow water environments [41]. Additionally, adult *P*. *astreoides* harboring *Symbiodinium* type A4 maintain their symbiotic associations throughout bleaching events [42].

The evidence for symbiont flexibility in adult corals for acclimation to environmental change is widespread, however, this process is largely unexplored as a mechanism of juvenile acclimation. Previous studies have examined the flexible symbioses of *Acropora spp*. and *Symbiodinium* during horizontal symbiont acquisition by aposymbiotic larvae, revealing the ability of different types of *Symbiodinium* to confer increased coral growth and thermotolerance at early life stages [21–23, 26]. Similar to their adult counterparts, juveniles inoculated with clade C *Symbiodinium* exhibited faster growth while those harboring clade D *Symbiodinium* exhibited increased thermotolerance [22, 23, 26]. In broadcast-spawning corals releasing aposymbiotic larvae, such as *Acropora spp*., the process of symbiont-acquisition is highly flexible, where juveniles at 3.5 years did not mirror the *Symbiodinium* populations in their adult counterparts [22, 25]. In contrast, in brooding corals that release symbiotic planula, such as *Stylophora pistillata*, *Symbiodinium* consortia in nearby pelagic planulae and juveniles (<3 cm width) largely mirrored adult *Symbiodinium* consortia [16].

Coral life-history strategies are thought to play a role in patterns of symbiotic associations where broadcast-spawned, aposymbiotic larvae are provided an advantage to acquire *Symbiodinium* consortia appropriate for their local environment but failure to acquire *Symbiodinium* consortia will result in death [27]. Aposymbiotic larvae and subsequent juveniles are often highly flexible in their symbiotic associations during early development, with specificity occurring later in development [22, 43]. In contrast, brooding corals vertically transmit *Symbiodinium* where they are phagocytized during embryo endoderm formation and tissue layer differentiation [15, 44]. Brooded, symbiotic planulae do not risk death from failed symbiont acquisition though they risk receiving symbionts suitable for the local environment that are not necessarily well-suited for dispersal to new environments [27]. The process of symbiont shuffling during vertical transmission of *Symbiodinium* to brooded planulae and during early growth of resulting juveniles is poorly understood but have profound implications for juvenile acclimation.

The goal of this study was to examine variations in *Symbiodinium* consortia throughout various life history stages of the brooding coral, *P*. *astreoides*. Symbiotic associations with colonies of *P*. *astreoides* from different depths were examined to determine if the consortia of *Symbiodinium* associated with shallow (10m) and upper mesophotic (30m) colonies (1) differ between depths, (2) change during early development, and (3) influence growth of juveniles under varying environmental conditions. Here we present insight into conserved symbiotic associations throughout vertical transmission in brooding corals, and the influence of environment on *Symbiodinium* consortia associated with early juveniles and their growth.

## Materials and methods

### Study site

**Fig 1:**
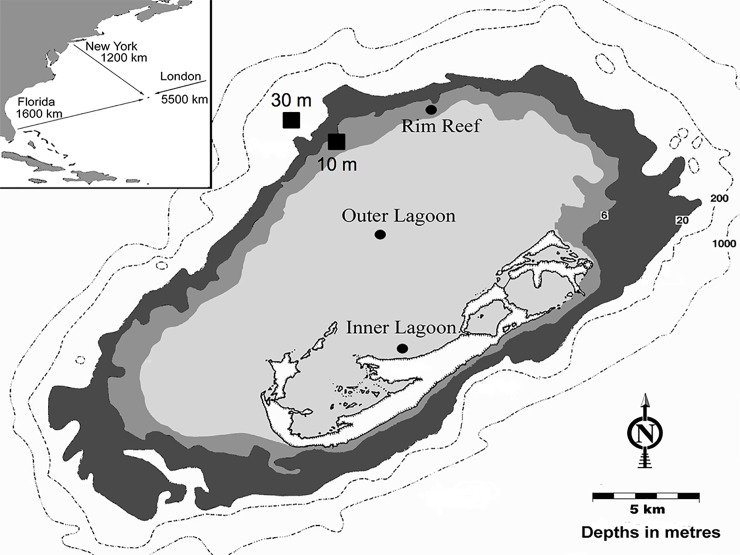
Map of shallow (10 m) and upper mesophotic (30 m) sampling sites in Bermuda. Sites were used for adult *Porites astreoides* colony collection and juvenile transplantation.

Collection of adult *Porites astreoides* and transplants of *P*. *astreoides* juveniles occurred at a shallow rim reef (10m, Hog Breaker) and an upper mesophotic (30m) reef in close horizontal proximity to each other (Fig 1; 10m: 32°45’77”N, 64°83’38”W; 30m: 32°48’80’N, 64°85’38”W). Adult *P*. *astreoides* colonies were collected from the aforementioned reefs on July 8 and August 5, 2015 following the guidelines of the Bermuda Institute of Ocean Sciences (BIOS) Collection and Experimental Ethics Policy’s “Limited Impact Research” policy (n = 10 per depth per month). Adult, planulae, and juvenile coral tissue samples were exported under CITES permits 15BM0010 and 15BM0014.

### Field and tissue collection methods

**Fig 2:**
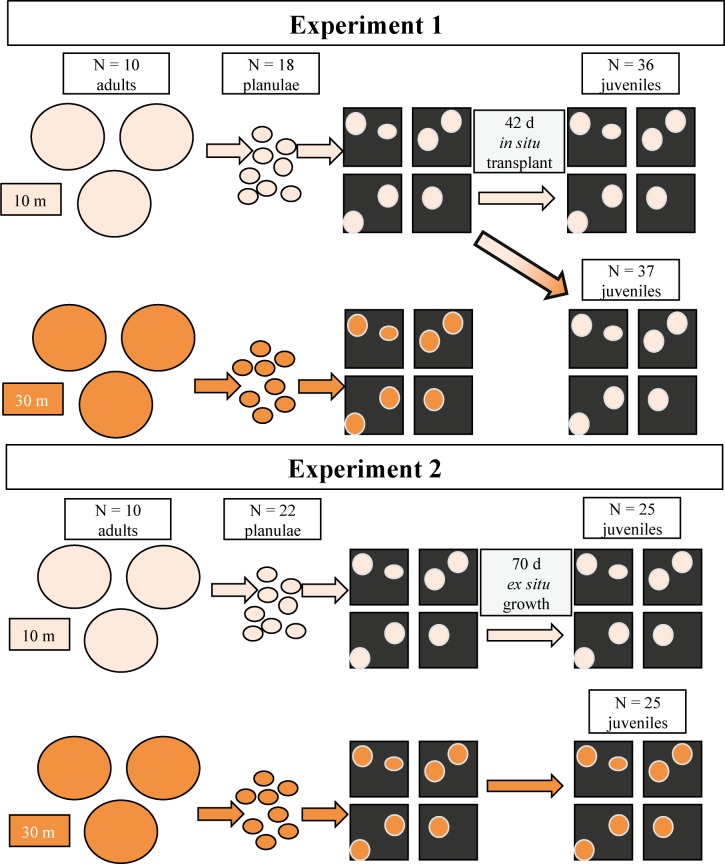
Flow-through of experimental design for experiments 1 and 2. In Experiment 1, *Symbiodinium* consortia were determined for shallow and upper mesophotic *Porites astreoides* adult colonies and planulae. Juveniles of shallow parental origin were reared *in situ* on the shallow and upper mesophotic reef and were examined for specific growth rates and *Symbiodinium* phylotype combinations. In Experiment 2, *Symbiodinium* consortia and specific growth rates were determined for shallow and upper mesophotic planulae and *ex situ* reared juveniles.

The field component of this study was composed of two experiments, the first conducted in July 2015 and the second in August 2015. In both *Experiment 1* (July) and *Experiment 2* (August), tissue was collected from adults, planulae, and juveniles to examine *Symbiodinium* consortia (Fig 2). However, in *Experiment 1*, juvenile spat from shallow adults were transplanted back to shallow and upper mesophotic reefs to examine *in situ* acclimation and juvenile growth rates. In *Experiment 2*, growth rates and *Symbiodinium* consortia were examined for juveniles produced from both shallow and upper mesophotic adults that were reared in *ex situ* laboratory conditions. See Fig 2 for a diagram of the experimental design.

#### Experiment 1

Adult *P*. *astreoides* colonies were collected using a hammer and chisel, placed in individual plastic bags, and were transported to BIOS in coolers containing natural seawater. Colonies were then placed in individual containers in a shallow, *ex situ*, flow-through seawater system and planulation events were monitored following the methods outlined in [45]. On each day that planulation occurred (in both July and August), a subset of newly released planulae were preserved in 95% ethanol for molecular symbiont analyses (>6 planulae coral^-1^ day^-1^). The remainder of the newly released planulae were pooled by reef zone and settlement was assessed by placing 100 planulae into 20, 0.5 L plastic settlement chambers with 125 μm mesh tops (to allow for water flow) each containing two preconditioned terracotta tiles. After 1 week, the number and position of newly settled juveniles were scored under a dissecting microscope. Juveniles were photographed and surface area was determined using the “free-hand” tool in ImageJ™. The tiles with newly settled juvenile corals were then transplanted onto the same shallow (n = 17 tiles) and upper mesophotic (n = 22 tiles) reefs from which adult colonies were collected (Fig 1). Tiles were placed in a cage covered in egg crate and placed parallel to the ocean floor. When transplanted on July 29, 2015 the water temperature was 28.3°C at the shallow reef and 26.7°C at the upper mesophotic reef. When samples were retrieved on August 28, 2015 the water temperature was 28.3°C at the shallow reef and 25.5°C at the upper mesophotic reef. Following the transplant, surface area of individual juvenile *P*. *astreoides* was measured as described above and pre- and post-transplant surface areas were used to calculate specific growth rates (% growth d^-1^). Juvenile *P*. *astreoides* (36 per transplant depth) were removed from the tiles and preserved in 95% ethanol. Before adult colonies were returned to the reef, ~3 cm^2^ of coral tissue was removed using a waterpik, zooxanthellae were concentrated by centrifugation, resuspended in 1 mL of 95% ethanol and stored at -20°C [46].

#### Experiment 2

In August, 10 adult *P*. *astreoides* colonies were collected from the same shallow and upper mesophotic sites sampled in *Experiment 1* (July) and planulae were collected in the manner described above. *Symbiodinium* were not isolated from these adult colonies, however, as outlined in the collection permit. Planulation was monitored and a subset of newly released planulae were preserved for molecular analyses with the remainder of planulae settled onto preconditioned terracotta tiles as in *Experiment 1*. After 1 week, newly settled juveniles from adults collected at both depths were photographed, sized using ImageJ™ and subsequently reared *ex situ* for 10 weeks in outdoor aquaria with flowing seawater at BIOS as described by [47]. Aquaria received continuous flowing seawater and were maintained under ambient light conditions and temperatures ranged from 24.0–29.4°C (S2 Fig). After 70 d, juveniles were photographed, sized, and specific growth rates were calculated as described above. Juvenile corals from both parental depths (n = 25 per depth) were then removed from the tiles and preserved in 95% ethanol for molecular analyses.

### Molecular analyses

DNA was extracted from ethanol preserved planulae, juveniles, and adult coral tissue samples using a modified DE-1 protocol on a Kurabo QuickGene-810 AutoGene (Holliston, MA), where the tissue was ground with a pestle in a 1.5 mL tube after the addition of lysis buffer MDT, or a Qiagen DNeasy Plant Mini Kit following the manufacturer’s protocols (Valencia, CA). DNA concentration for each sample was spectrophotometrically quantified using a Thermo Scientific NanoDrop Lite spectrophotometer (Waltham, MA). For all samples, 1 μL of undiluted DNA was amplified in a 25 μL reaction with the following reagents: 17.75 μL dH_2_O, 2.5 μL New England Biolabs Taq Buffer (Ipswich, MA), 1 μL MgCl_2_, 1 μL 10 mM ITS-DINO (Table 1; [48]), 1 μL 10 mM ITS2rev2 (Table 1; [48]), 0.625 μL dNTP (10 mM each; New England Biolabs), and 0.125 μL Taq Polymerase (New England Biolabs). The concentration of DNA in each reaction ranged from 0.5–50.1 ng μL^-1^. The primers were designed to amplify the ITS2 region of all known *Symbiodinium* phylotypes [48]. DNA was amplified using a MJ Research Peltier Thermal Cycler (PTC-200; Ramsey, MN) with an initial denaturation at 95°C for 5 min, followed by 36 cycles of 94°C for 1 min, 57°C for 45 s, and 72°C for 30 s and a final extension at 72°C for 10 min. PCR products were visualized on a 2.0% Lonza NuSieve GTG Agarose (Walkersville, MD) gel in 0.5x Tris-Acetate-EDTA (TAE) buffer and run for 90 min at 80 V [49]. For samples that yielded two PCR products (upper band *Porites astreoides* 389 bp, lower band *Symbiodinium sp*. 313 bp), the smaller PCR product was gel-purified (Monarch Kit, New England Biolabs), re-amplified, and visualized as described above.

**Table 1:** PCR primers used in this study.

Primer name	Region	Sequence 5’-3’	Reference
ITS-DINO	ITS2 forward	GTGAATTGCAGAACTCCGTG	Pochon *et al*. 2001
ITS2rev2	ITS2 reverse	CCTCCGCTTACTTATATGCTT	Pochon *et al*. 2001
Clade A ITS2	ITS2 (clade specific)	ATGGCACTGGCATGC	Arif *et al*. 2014
Clade B ITS2	ITS2 (clade specific)	ATTGCTGCTTCGCTTTCC	Arif *et al*. 2014
Clade C ITS2	ITS2 (clade specific)	TGCTTAACTTGCCCCAAC	Arif *et al*. 2014
Degenerate reverse primer	ITS2 (clade specific)	TCWCYTGTCTGACTTCATGC	Arif *et al*. 2014

Both strands of the PCR products were sequenced directly using single-pass Sanger sequencing services from Macrogen (Cambridge, MA) and assembled into individual contigs using CodonCode Aligner v3.5.6 (CodonCode Corporation, Centerville, MA). All sequences were compared with sequences in the non-redundant nucleotide collection at NCBI using BLASTN [50] and were identified as *Symbiodinium* type A4. Sequences were aligned using the ClustalW algorithm in MacVector v14.5.3 (MacVector, Inc, Apex, NC) and a consensus sequence was created, which was identical to *Symbiodinium* phylotype A4 (CCMP2456, LK934674.1) deposited in NCBI. A pairwise percent similarity matrix was generated (ignoring gaps) using Geneious v9.1.3 [51]. All sequences and sample information are publically available via Clark University’s Digital Commons (http://commons.clarku.edu/facultyworks/24/) and a representative sequence was deposited on Genbank (accession number KY273433).

ITS2 PCR products were used to screen for the presence/absence of phylotypes A, B, and C using 1 μL of degenerate reverse primer (10 mM) and 1 μL of forward primers (10 mM) designed to amplify phylotype A, B, or C (Table 1; [52]). PCR products representing each phylotype were visualized on a 2.0% Lonza NuSieve GTG Agarose gel in 1x Tris-borate-EDTA (TBE) buffer and run for 120 min at 80 V [49]. Samples were scored as positive for the individual phylotypes based on the presence of appropriately sized PCR product (phylotype A 100 bp, phylotype B 180 bp, and phylotype C 210 bp). For confirmation, PCR products were gel purified using a New England Biolabs Monarch kit, amplified, and sequenced directly using single-pass Sanger sequencing services from Macrogen (Cambridge, MA). Representative sequences for phylotypes B and C were deposited on Genbank (accession numbers KY273434-35, respectively).

### Statistical analyses

Chi-squared analyses were used to test for differences in the frequency of occurrence of *Symbiodinium* phylotypes among adult, planulae, and juvenile *P*. *astreoides*. A two-by-c Tukey-type multiple comparisons of proportions was used to determine whether the proportion of corals harboring only *Symbiodinium* phylotype A or *Symbiodinium* phylotype A in combination with other phylotypes varied independently in different life stages [53]. A two-factor Analysis of Variance (ANOVA) was used to test for differences in the specific growth rates (% growth) of *in situ* transplanted juveniles using symbiont phylotype and transplant depth as factors. Growth rates (% growth d^-1^) did not meet assumptions of parametric statistics so a randomized two-factor ANOVA with 10,000 permutations was used [54]. A randomized one-factor ANOVA with 10,000 permutations [54] and pairwise posthoc comparison using Tukey and Kramer (Nemenyi) test with Tukey-Dist approximation was used to test for differences in the specific growth rates (% growth d^-1^) of juveniles reared *in situ* at different depths and *ex situ* of different parental depths. Statistical analyses were performed in R [55].

## Results

DNA from adult corals, planulae, and juveniles were initially amplified using PCR primers that amplify the ITS2 region of all known *Symbiodinium* phylotypes [48]. All samples (n = 210) were identified as *Symbiodinium* type A4 and had an average percent similarity of 99.96% ± 0.02% (standard error) to the reference *Symbiodinium* type A4 sequence (CCMP2456, LK934674.1) deposited in NCBI. Percent similarity to the reference sequence ranged from range of 98 and 100%.

### *Symbiodinium* consortia across depths

**Fig 3:**
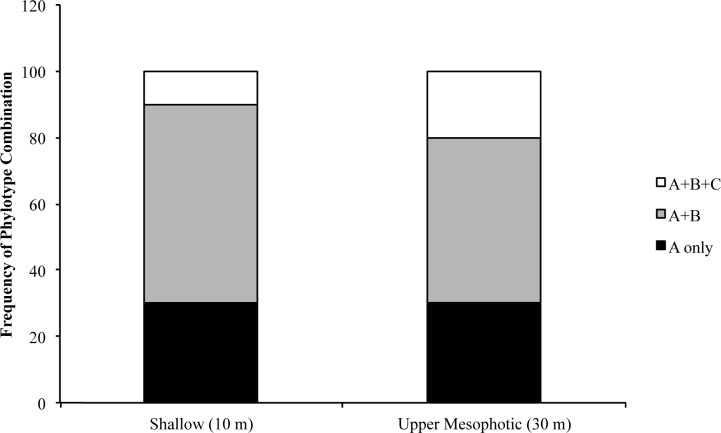
*Symbiodinium* phylotype composition of field-collected shallow and upper mesophotic adult *Porites astreoides*. There was no significant difference in *Symbiodinium* phylotype frequency between depths.

Although *Symbiodinium* phylotype A4 was the most abundant phylotype in *P*. *astreoides*, *Symbiodinium* phylotypes B and C were detected in nested PCR reactions using clade specific primers. Adults from both shallow (n = 10) and upper mesophotic (n = 10) reefs had three symbiont combinations: only phylotype A, phylotypes A and B, and phylotypes A, B, and C (Fig 3). The combination of *Symbiodinium* phylotypes A and B was detected at the highest frequency in adults from both shallow and upper mesophotic reefs and the distribution of phylotypes did not vary with depth (χ^2^ = 0.42, d.f. = 2, p = 0.81).

### Vertical transmission of *Symbiodinium* consortia

**Fig 4:**
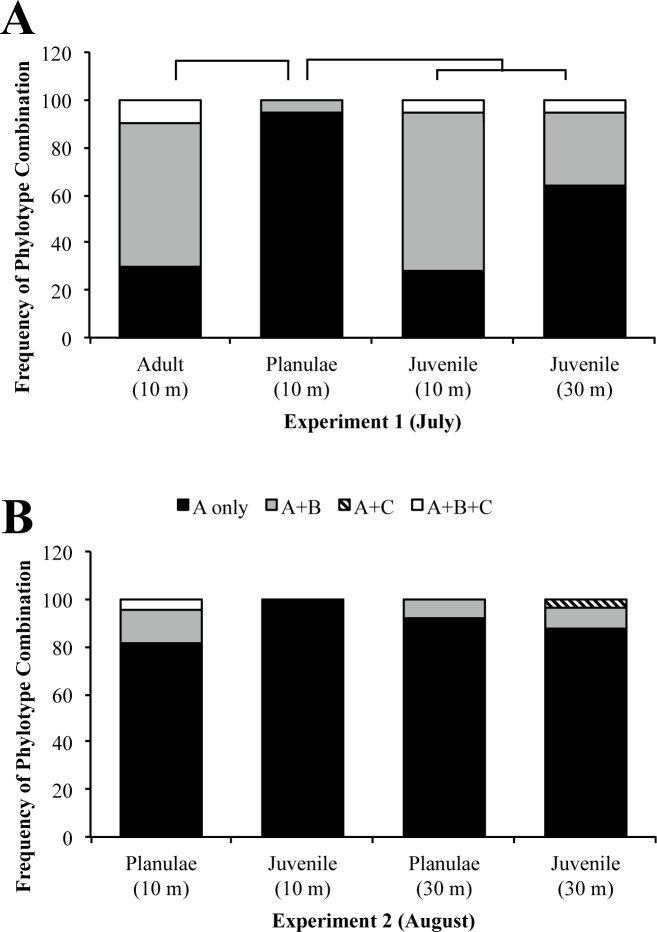
***Symbiodinium* phylotype composition in various *Porites astreoides* life stages reared *in situ* (A) and *ex situ* (B).** (A) *Symbiodinium* phylotype composition of shallow *P*. *astreoides* adults, their brooded planulae, and newly settled juveniles reared *in situ* on shallow (10 m) and upper mesophotic (30 m) reefs. *Symbiodinium* phylotype frequencies differed significantly between (1) adults and planulae, (2) planulae and juveniles transplanted to the shallow reef, (3) planulae and juveniles transplanted to the upper mesophotic reef, and (4) between juveniles transplanted to the different depths. There was no difference in *Symbiodinium* phylotype frequencies between adults and juveniles transplanted to different depths. (B) *Symbiodinium* phylotype composition of planulae and *ex situ*, reared juvenile *P*. *astreoides*. No difference was found in *Symbiodinium* phylotype frequency of (1) planulae or juveniles from different parental depths or (2) between planulae and juvenile.

*Symbiodinium* phylotype frequencies varied significantly by developmental stage for shallow adult *P*. *astreoides*, the planulae they released, and resulting early juveniles (χ^2^ = 230.18, d.f. = 6, p = 7.02e-47). A Tukey-type multiple comparisons of proportions of corals harboring only *Symbiodinium* phylotype A versus *Symbiodinium* phylotype A in combination with other phylotypes was used to determine variation between the specific life stages (Table 2; [53]). *Symbiodinium* phylotype frequencies were significantly different in shallow adult *P*. *astreoides* and the planulae they released (Table 2; q = 5.06). Shallow (n = 10) adult colonies had three symbiont compositions: phylotype A only, phylotypes A and B, and phylotypes A, B, and C (Fig 4). This pattern was not observed in their planulae (n = 18) in which the majority of planulae contained only phylotype A and with the rest of the planulae containing both A and B phylotypes (Fig 4). *Symbiodinium* phylotype C was not detected in the planulae collected for analyses.

**Table 2:** Tukey-type multiple comparison of proportions of *Symbiodinium* phylotype frequencies in *P*. *astreoides* life stages.

Comparison	p'B-p'A	SE	q	Critical q_0.05,_∞_,4_
Adult vs. planulae	39.59	7.83	**5.06**	3.63
Planulae vs. juveniles transplanted to shallow reef	41.73	5.78	**7.22**	3.63
Planulae vs. juveniles transplanted to upper mesophotic reef	21.03	5.78	**3.64**	3.63
Shallow juveniles vs. upper mesophotic juveniles	20.70	4.74	**4.37**	3.63

### *Symbiodinium* consortia in juvenile corals reared *in situ* vs. *ex situ*

After being transplanted to the shallow and upper mesophotic reef, *Symbiodinium* phylotypes A, B, and C were detected in the juvenile *P*. *astreoides* (Fig 4). The majority of juveniles transplanted to the shallow reef harbored phylotypes A and B whereas juveniles transplanted to the upper mesophotic reef harbored only phylotype A (Fig 4). Juveniles transplanted to the shallow reef and upper mesophotic reefs had significantly different *Symbiodinium* phylotype frequencies than the source population of shallow planulae (Fig 4; Table 2; q = 7.22; q = 3.64). *Symbiodinium* phylotype frequencies also varied significantly between the juveniles transplanted to different depths (Fig 4; Table 2; q = 4.37). In addition, juveniles transplanted to both depths had similar *Symbiodinium* phylotype frequencies as the shallow adult colonies (shallow, q = 0.30; upper mesophotic, q = 2.62).

**Fig 5:**
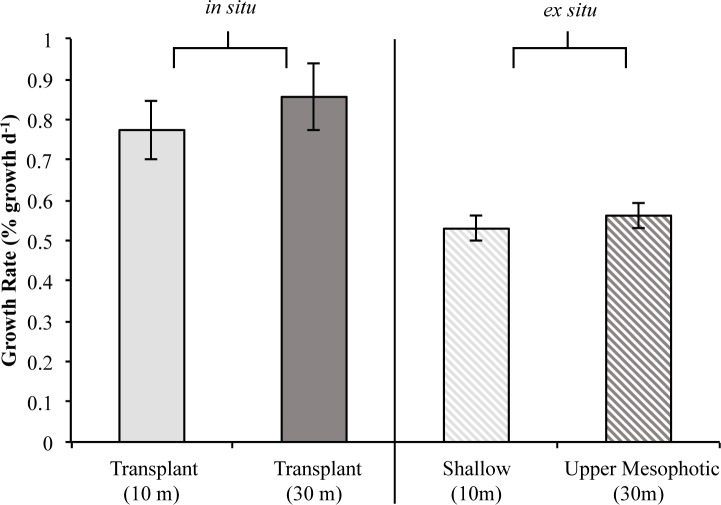
Specific growth of *in situ* and *ex situ* reared *Porites astreoides*. Specific growth of juvenile *P*. *astreoides* reared on shallow (10 m) and upper mesophotic (30 m) reefs (*in situ*) did not differ using transplant depth and *Symbiodinium* phylotype combinations as factors (left). Specific growth of shallow and upper mesophotic juvenile *P*. *astreoides* reared in outdoor aquaria (*ex situ*) also did not differ (right). Both treatments of *in situ* reared juveniles (left) had significantly higher growth rates than both treatments of *ex situ* reared juveniles (right). Shaded bars represent mean specific growth (% growth d^-1^) ± SE.

There was no significant difference in the average specific growth rates of juveniles transplanted onto the shallow and upper mesophotic reefs (t-test, p = 0.46). Juveniles transplanted to the shallow reef had an average growth rate of 0.77±0.07 (% growth d^-1^ ± standard error) whereas juveniles transplanted to the upper mesophotic reef had an average growth rate of 0.86±0.08 (Fig 5). Additionally, there was no difference in juvenile growth with the factors *Symbiodinium* phylotype composition (F = 0.022, p = 0.98) and transplant depth (F = 0.003, p = 0.96), or their interaction (F = 0.524, p = 0.58). Likewise, no difference was found in average percent mortality of juveniles transplanted to the shallow (40.49%±9.40%; average ± standard error) and upper mesophotic (35.37%±8.22%) reefs (t-test, p = 0.68).

In *Experiment 2* (August), there was no difference in *Symbiodinium* phylotype frequencies between shallow and upper mesophotic planulae (χ^2^ = 1.52, d.f. = 2, p = 0.47). The majority of shallow and upper mesophotic planulae harbored only phylotype A with the remaining harboring phylotypes A and B (Fig 4). All shallow juveniles harbored exclusively phylotype A; whereas the majority of upper mesophotic juveniles harbored only phylotype A with the remaining few harboring either phylotypes A and B, or phylotypes A and C (Fig 4). In addition, *Symbiodinium* phylotype frequencies varied but were not significantly different when comparing planulae and juveniles from either shallow (χ^2^ = 4.97, d.f. = 2, p = 0.083) or upper mesophotic parental origin (χ^2^ = 0.98, d.f. = 2, p = 0.61). There was no difference in *Symbiodinium* phylotype frequencies or growth rates between shallow and upper mesophotic juveniles raised in *ex situ* outdoor aquaria for 10 weeks (Fig 4; Fig 5; χ^2^ = 3.19, d.f. = 2, p = 0.20). Juveniles of shallow parental origin had an average growth rate of 0.53±0.03 (% growth d^-1^ ± standard error) and those of upper mesophotic parental origin had an average growth rate of 0.56±0.03 (Fig 5). In addition, there was no significant difference in average percent mortality of shallow (62.63%±6.33%; average ± standard error) and upper mesophotic (74.22%±5.40%) juveniles reared *ex situ* in outdoor aquaria (t-test, p = 0.17).

There was no significant difference in *Symbiodinium* phylotype frequency between shallow planulae from July and August, with the majority of samples only harboring phylotype A (χ^2^ = 1.65, d.f. = 2, p = 0.44; Fig 4). However, there was a difference in *Symbiodinium* phylotype frequency between juveniles reared on reefs compared with those reared *ex situ* in outdoor aquaria at BIOS. Juveniles reared on either shallow or upper mesophotic reefs had an increased presence of *Symbiodinium* phylotypes B and C compared with juveniles reared in outdoor aquaria. *Symbiodinium* phylotype frequencies varied significantly between juveniles of shallow parental origin reared on a shallow reef and juveniles of shallow parental origin grown *ex situ* shallow water conditions (χ^2^ = 31.47, d.f. = 2, p = 1.47e-7; Fig 4). There was a difference in *Symbiodinium* phylotype frequency between juveniles of shallow parental origin reared on an upper mesophotic reef and upper mesophotic juveniles reared in *ex situ* shallow water conditions, (χ^2^ = 5.51, d.f. = 3, p = 0.057).

There was a significant difference in the specific growth (% growth d^-1^) of juveniles reared in different environments (*in situ*, [shallow and upper mesophotic reefs] vs. *ex situ*, [outdoor aquaria]; % growth d^-1^) using experimental group as a factor (F = 10.1, d.f. = 3, p = 2.05e-6). Pairwise comparisons using Tukey & Kramer (Nemenyi) tests with Tukey-Dist approximations were used to compare specific growth of juvenile *P*. *astreoides* transplanted to shallow and upper mesophotic reefs (*in situ*) and shallow and upper mesophotic juveniles reared in outdoor aquaria (*ex situ*). Juveniles transplanted to either the shallow or upper mesophotic reef grew significantly faster than shallow juveniles (p = 0.003, p = 7.10e-6; respectively) and upper mesophotic juveniles (p = 0.011, p = 5.30e-5; respectively) reared in outdoor aquaria (Fig 5).

## Discussion

### Effects of depth on *Symbiodinium* consortia

Physiological diversity among *Symbiodinium* phylotypes supports coral distribution across a wide variety of environments ranging from those with high light levels [41, 56, 57] to those adapted to the low-light and temperatures associated with the lower mesophotic (60-100m; [58]). This study examined *Symbiodinium* consortia in *Porites astreoides* adults over a depth gradient where environmental factors such as temperature, nutrients, and light vary with depth. In Bermuda, seawater temperatures are warmer and more variable on shallow reefs compared with deeper reefs [59]. In addition, concentrations of nitrate and nitrite were found to be higher at shallow depths than deeper depths (10 and 45 m, respectively; [59]). Offshore of Bermuda, the light extinction coefficient is estimated to range between 0.025–0.55 m^-1^ during July and August, resulting in a gradual decrease in light between 10 and 30 m depth [60]. Despite these variations, there was no difference in the frequencies of *Symbiodinium* phylotypes in adult *P*. *astreoides* residing at 10 and 30 m (Fig 3). In contrast, variations in the distribution of *Symbiodinium* phylotypes associated with adult *P*. *astreoides* have been observed along depth gradients in several locations throughout the Caribbean [9, 30, 61, 62]. The lack of differentiation in *Symbiodinium* consortia in *P*. *astreoides* in our study may reflect less steep environmental gradients in Bermuda compared with regions in the Caribbean. Additionally, shallow and upper mesophotic *P*. *astreoides* in Bermuda have high levels of genetic connectivity and are thought to belong to a regionally isolated and locally sustained coral population which does not exhibit symbiont depth zonation [9].

We detected greater diversity in *Symbiodinium* than reported in other studies of *P*. *astreoides* in Bermuda [9, 40, 63]. The increased diversity in *Symbiodinium* observed here may be a result of differences in sampling locations. In this study, *P*. *astreoides* were collected from a northern rim reef, which has higher coral cover and diversity [64] relative sites selected in previous studies [9]. High coral density and diversity may lead to increased *Symbiodinium* diversity and availability in the water column [65], which in turn, may translate to the increased diversity of phylotypes observed in *P*. *astreoides* adults at these locations. Alternatively, the increased diversity observed in our study may reflect the nested PCR approach used to screen for specific *Symbiodinium* phylotypes. Recent studies have shown that amplification and direct sequencing or DGGE analyses of *Symbiodinium* ITS2 are unable to detect phylotypes that are in low abundance (e.g. less than 5–10% of the population; [35, 66, 67]). In this study, the first round of PCR used ITS2 primers that amplify all known *Symbiodinium* phylotypes and may have increased the abundance of phylotypes B and C amplicons to a level that was detected in the second round of PCR using clade-specific primers. Future studies that take advantage of quantitative PCR methods will be important to quantify the relative abundances of the different phylotypes.

### Vertical transmission of *Symbiodinium* and variations in early development

Though the presence of phylotypes B and C in combination with phylotype A was found in the majority of adult corals, this pattern was not observed in planulae, of which all harbored phylotype A with the majority having only phylotype A (Fig 3). The decrease in diversity of *Symbiodinium* associated with *P*. *astreoides* planulae may be attributed to unique microenvironments and energetic requirements associated with brooding. *P*. *astreoides* eggs develop and planulae are brooded throughout the mesenteries, which may provide unique microenvironments within the corals [15, 68, 69]. The energy demands of brooded planulae are thought to be derived from maternal lipids and protein reserves rather than *Symbiodinium*, thus exogenous uptake or internal proliferation of non-dominant *Symbiodinium* may occur later in development after transitions in the metabolic interactions between the host and symbiont [70]. In the Red Sea, Byler *et al*. [16] found that the dominant *Symbiodinium* type in adult colonies of the brooding coral *Stylophora pistillata* to be vertically transmitted to planulae. This pattern occurred throughout the bathymetric distribution of *S*. *pistillata* in which *Symbiodinium* phylotypes varied with depth [16].

The increase in *Symbiodinium* phylotypes observed in reef-reared juveniles compared to their planulae suggests that horizontal *Symbiodinium* transmission during early development in brooding corals (Fig 4). When transplanted to the shallow reef, juveniles appeared to mirror shallow adult *P*. *astreoides Symbiodinium* phylotype combinations whereas juveniles transplanted to the upper mesophotic reef appeared to be in the process of mirroring mesophotic adult *P*. *astreoides Symbiodinium* consortia (Fig 3, Fig 4). It is also possible that the increase in *Symbiodinium* phylotype B and C detected in juvenile *P*. *astreoides* compared to the planulae may reflect the increase in the amount tissue used for molecular analyses; the increase in biomass may have allowed for the detection of previously undetectable phylotypes. Alternatively, the presence of phylotypes may change due to differential metabolic needs of juvenile *P*. *astreoides* during development [70].

### Effects of environmental condition on symbiotic associations and juvenile growth

Although frequencies of *Symbiodinium* phylotype combinations in juvenile *P*. *astreoides* varied between depths, there was no significant difference in growth rates (Fig 5). This suggests that when present, phylotypes B and C behaved as background symbionts having a transitory influence on coral growth [71]. Growth may have been compensated by variation in environmental factors associated with the transplant depths where juveniles transplanted to the shallow reef were exposed to higher nutrient levels, higher light, and warmer seawater temperatures than those juveniles transplanted to the upper mesophotic. Thus a tradeoff may exist for juvenile coral growth under varying environmental conditions at the different depths which may have allowed for similar growth rates.

Juveniles reared in *ex situ* outdoor aquaria had significantly different frequencies of *Symbiodinium* phylotype combinations than those reared in *in situ* reef environments where the majority of juveniles hosted only phylotype A (Fig 4). Additionally, there was no difference in growth of juvenile *P*. *astreoides* from both parental depths (Fig 5). In the *ex situ* outdoor aquaria at BIOS (<1m), it may be advantageous for juvenile *P*. *astreoides* to only host *Symbiodinium* type A4 as they produce UV-protecting compounds [56, 57] in addition to physiological pathways associated with increased photoprotection [41]. These physiological traits may provide the coral competitive advantage in extreme shallow water environments. Thus, the lack of *Symbiodinium* diversity may be due to continuously high light levels experienced in the outdoor aquaria promoting selection for photo-protective symbiont types [41]. Alternatively, the reduced frequencies of phylotypes B and C may indicate there is greater symbiont availability in the reef environment due to the presence of adult colonies and sediments exchanging *Symbiodinium* into the water column. Nitschke *et al*. [65] found that juvenile *Acropora spp*. had higher *Symbiodinium* densities when in the presence of adult colonies and sediments as a result of coral expulsion of *Symbiodinium* into the water column. The higher frequencies of *Symbiodinium* phylotypes B and C in the *in situ* reared juveniles compared with those reared *ex situ* in aquaria may be due to increased symbiont availability on the reef in comparison to the aquaria. In addition to having significantly different frequencies of *Symbiodinium* phylotype combinations, the juveniles reared in outdoor aquaria grew significantly slower than those reared on the reef (Fig 4, Fig 5). This is likely due to the differences in the environments as juveniles reared in the outdoor aquaria were exposed to ambient light and temperature, making it a potentially a stressful environment in comparison to the reef.

To our knowledge, this study is among the first to examine *Symbiodinium* consortia through vertical transmission and early growth of a brooding coral. We document greater diversity of *Symbiodinium* phylotypes associated with *P*. *astreoides* throughout its depth distribution in Bermuda than previously observed [9]. However, only the dominant symbiont, *Symbiodinium* phylotype A4 was vertically transmitted to the majority of planulae. When juveniles were reared at different depths on the reef, the *Symbiodinium* consortia appeared to transition and mirror *Symbiodinium* phylotype combinations associated with adult *P*. *astreoides*. Juveniles transplanted to different depths had significantly different frequencies of *Symbiodinium* phylotype combinations but no difference in growth, suggesting potential for selection during juvenile development post transplantation. In contrast, *P*. *astreoides* juveniles reared in outdoor aquaria did not transition to mirror *Symbiodinium* phylotype combinations associated with adult reef *P*. *astreoides* and had slower growth rates than their reef-reared counterparts. By comparing growth and symbiotic associations of *ex situ* and *in situ* reared juveniles, this study highlights the impact of habitat type on growth and *Symbiodinium* consortia of juvenile corals. This is crucial for past and future interpretation of research involving juvenile corals reared in various environments. Moreover, these results document patterns of horizontal transmission of symbionts from parental *P*. *astreoides* colonies to their planulae, and variability in *Symbiodinium* consortia across developmental stages of juveniles reared in different environments. Taken together, this study indicates that while the relationship between *P*. *astreoides* and *Symbiodinium* type is conserved across reproduction, flexibility in the associated consortia during development may enable adaptation to various environments, which may aid the long-term success of the species under changing conditions.

## Supporting information

S1 Fig Juvenile *Porites astreoides* before and after transplantation to a shallow (10 m) and upper mesophotic (30 m) reef for 42 d. Photographs are property of Kevin H. Wong (used with permission).
(EPS)

S2 Fig Daily average water temperature (°C) of outdoor aquaria. Aquaria were used to rear shallow and upper mesophotic juvenile *Porites astreoides*.
(EPS)
